# Impact of trans-stent gradient on outcome after PCI: results from a HAWKEYE substudy

**DOI:** 10.1007/s10554-022-02708-7

**Published:** 2022-08-22

**Authors:** Andrea Erriquez, Barry F. Uretsky, Salvatore Brugaletta, Giosafat Spitaleri, Enrico Cerrato, Giorgio Quadri, Marco Manfrini, Graziella Pompei, Davide Scancarello, Michele Trichilo, Federico Marchini, Serena Caglioni, Roberta Campana, Andrea Marrone, Carlo Penzo, Carlo Tumscitz, Matteo Tebaldi, Filippo Maria Verardi, Antonella Scala, Gianluca Campo, Simone Biscaglia

**Affiliations:** 1 Cardiovascular Institute, Azienda Ospedaliero-Universitaria di Ferrara, S. Anna, Via Aldo Moro 8, 44124 Cona, FE Italy; 2 Central Arkansas VA Health System, Little Rock, AR USA; 3 Cardiovascular Clinic Institute, Institut d’Investigacions Biomèdiques August Pi i Sunyer (IDIBAPS), University Hospital Clínic, Barcelona, Spain; 4 San Luigi Gonzaga University Hospital, Orbassano and Infermi Hospital, Rivoli, Turin Italy; 5 Maria Cecilia Hospital, GVM Care & Research, Cotignola, RA Italy

**Keywords:** Angiography-based fractional flow reserve, Trans-stent gradient, Outcome, Percutaneous coronary intervention, Vessel-oriented composite endpoint

## Abstract

To test whether quantitative flow ratio (QFR)-based trans-stent gradient (TSG) is associated with adverse clinical events at follow-up. A post-hoc analysis of the multi-center HAWKEYE study was performed. Vessels post-PCI were divided into four groups (G) as follows: G1: QFR ≥ 0.90 TSG = 0 (n = 412, 54.8%); G2: QFR ≥ 0.90, TSG > 0 (n = 216, 28.7%); G3: QFR < 0.90, TSG = 0 (n = 37, 4.9%); G4: QFR < 0.90, TSG > 0 (n = 86, 11.4%). Cox proportional hazards regression model was used to analyze the effect of baseline and prognostic variables. The final reduced model was obtained by backward stepwise variable selection. Receiver operating characteristic (ROC) was plotted and area under the curve (AUC) was calculated and reported. Overall, 449 (59.8%) vessels had a TSG = 0 whereas (40.2%) had TSG > 0. Ten (2.2%) vessel-oriented composite endpoint (VOCE) occurred in vessels with TSG = 0, compared with 43 (14%) in vessels with TSG > 0 (p < 0.01). ROC analysis showed an AUC of 0.74 (95% CI: 0.67 to 0.80; p < 0.001). TSG > 0 was an independent predictor of the VOCE (HR 2.95 [95% CI 1.77–4.91]). The combination of higher TSG and lower final QFR (G4) showed the worst long-term outcome while low TSG and high QFR showed the best outcome (G1) while either high TSG or low QFR (G2, G3) showed intermediate and comparable outcomes. Higher trans-stent gradient was an independent predictor of adverse events and identified a subgroup of patients at higher risk for poor outcomes even when vessel QFR was optimal (> 0.90).

**Supplementary Information:**

The online version contains supplementary material available at 10.1007/s10554-022-02708-7.

## Introduction

Percutaneous coronary revascularization (PCI) with stent implantation has significantly improved symptoms and clinical outcomes in patients with coronary artery disease (CAD) [1–4]. Yet, one out of four patients may experience residual angina/ischemia after angiographically “successful” PCI [5]. This finding may be due to either overlooked non-epicardial lesion (i.e.: microvascular dysfunction) or to unrecognized and untreated residual epicardial disease [6], either residual diffuse disease, an undiagnosed focal lesion outside the stented segment, stent underexpansion or undersizing or a combination of these abnormalities [5, 7, 8].

Limitations in coronary blood flow after angiographically optimized PCI may be evaluated by multiple modalities. The most well studied invasive modality is fractional flow reserve (FFR) using a pressure wire in the coronary artery. Multiple studies have shown an associated between FFR after intervention and long-term outcomes [9–15] with the lowest stratum of FFR showing the highest level of major adverse cardiac events (MACE) including cardiac death, myocardial infarction (MI), and target vessel revascularization (TVR). In most studies TVR dominated MACE events.

A promising newer method to measure FFR without a wire in the coronary is quantitative functional ratio (QFR). This method computes virtual fractional flow reserve (FFR) throughout a vessel utilizing coronary angiography [7, 8]. Previous studies have shown excellent correlation with invasively measured FFR [16–18]. The prospective multicenter HAWKEYE study demonstrated that long-term prognosis was associated with final QFR after PCI.

It has previously been shown in multiple studies that an underexpanded stent as measured by intravascular imaging increases the risk of long-term adverse clinical outcomes [19–23]. Further the extent of trans-stent FFR change (trans-stent gradient or TSG) has been shown to correlate with residual ischemia after PCI [24]. Further the severity of TSG by invasive FFR may be related to long-term outcomes [25].

The purpose of the present study, utilizing the HAWKEYE population [7], was to test whether TSG measured by QFR relates to adverse clinical events in follow-up in consecutive patients undergoing myocardial revascularization with successful stent implantation and to determine if its addition may improve outcome prognosis.

## Materials and methods

### Study design

The multicenter, prospective HAWKEYE (Angio-based Fractional Flow Reserve to Predict Adverse Events After Stent Implantation) study investigated the ability of QFR **(**Medis Medical Imaging Systems, Leiden, the Netherlands) to discriminate adverse events after successful PCI [7]. The study was conducted at seven centers in two countries (Italy and Spain) in accordance with the ethical principles of the Declaration of Helsinki. Patients were informed that their participation was voluntary, and all gave informed written consent. Methods and main results of the study have been reported [7]. We performed a post-hoc analysis to determine the value of QFR-TSG, that is, the QFR gradient across the stented segment of the target vessel, in predicting long-term outcomes and its relative importance to post-PCI QFR.

### Study patients

Patients ≥ 18 years who underwent PCI were eligible for the acquisition of the projections for QFR computation if (i) PCI was successful, (ii) complete revascularization was achieved, and (iii) second-generation drug-eluting stents (DES) were implanted. Successful PCI was defined as residual stenosis < 20% by visual estimation and final Thrombolysis. In Myocardial Infarction (TIMI) flow 3. Indication for PCI was left to the operator’s discretion based on clinical and angiographic data. Exclusion criteria were (i) ST-segment elevation myocardial infarction (STEMI), (ii) clinical or angiographic features limiting QFR computation (left main or ostial right coronary artery, previous coronary artery bypass graft, atrial fibrillation, ongoing ventricular arrhythmias, and persistent tachycardia (> 100 bpm), (iii) inability to provide consent, or (iv) life expectancy < 1 year.

### Study procedures

Invasive coronary angiography and PCI were performed following best local practices. Post-dilatation with non-compliant balloon was strongly encouraged but not mandated. At the end of the procedure, two angiographic projections for each vessel treated with PCI were acquired for QFR computation. Angiographic projections were acquired after nitroglycerine (100–200 µg) administration at 15 frames/sec during a single injection of 6 ml of contrast medium at a flow of 4 ml/sec and at a pressure of 300 psi, using a power injector system. Angiographic projections were at least 25° apart and aimed to provide minimal vessel foreshortening and vessel overlap. In agreement with previous studies [7, 26, 27], operators followed a table of recommended projection angles.

### Quantitative flow ratio and trans-stent gradient calculation

QFR computation was performed offline, using the software package QAngio XA 3D (Medis Medical Imaging System, Leiden, the Netherlands) in agreement with the step-by-step procedure validated in previous studies [7, 26, 27]. In the present analysis, we considered the contrast QFR value that was calculated in the entire vessel, starting from the most proximal available segment until its diameter became less than 1.5 mm. For the determination of QFR-TSG we positioned the proximal (p) and distal (d) marker for lesion QFR computation at the proximal and distal edges of the stent. We therefore obtained a lesion QFR that was equal to the QFR value measured between the proximal (p) and distal (d) edge of the stent. The QFR-TSG value was then calculated by subtracting from 1 the value of above mentioned lesion QFR in order to obtain the numerical physiological impact in terms of QFR across the stented segment (see supplemental material).

Finally, we divided vessels into four groups, on the basis of QFR-TSG and post-PCI QFR values: group 1 with QFR post-PCI ≥ 0.90 and QFR-TSG < 0.01; group 2 with QFR post-PCI ≥ 0.90 and QFR-TSG ≥ 0.01; group 3 with QFR post-PCI < 0.90 and QFR-TSG < 0.01 and group 4 with QFR post-PCI < 0.90 and QFR-TSG ≥ 0.01.

The TSG of 0.00 was chosen as cut-off value as it was the median TSG value (see Results). QFR computation was performed by the core laboratory of the University Hospital of Ferrara. Two independent operators (AE and AS), certified for QFR computation and blinded to outcome, performed the QFR and TSG computation.

### Quantitative coronary angiography and SYNTAX score calculation

Quantitative coronary analysis (QCA) and Synergy Between Percutaneous Coronary Intervention with Taxus and Cardiac Surgery (SYNTAX) score calculation was performed in the core laboratory of the University Hospital of Ferrara by operators (AE and AS) blinded to outcome. QCA was performed with validated software (CAAS II, Pie Medical System, Maastricht, the Netherlands). The following QCA values were measured before and after PCI: reference vessel size, lesion length and percent diameter stenosis (%DS) [13]. The above-mentioned values were measured at the level of the stented segment [13]. The SYNTAX score was calculated from the baseline coronary angiography before PCI. For each patient, by scoring all coronary lesions with stenosis diameter ≥ 50% in vessels ≥ 1.5 mm, the baseline score value was calculated using the SYNTAX score algorithm available online.

### Data collection and follow-up

Patient demographic data, cardiovascular risk factors, clinical diagnoses, and procedural details were recorded at the time of the PCI. Source data were collected on-line using dedicated electronic case report forms. Study angiograms were anonymized and submitted to core laboratory of the University Hospital of Ferrara. Clinical follow-up was performed at 30 days, and then every six months. Follow-up was censored at the end of November 2018 or at the time of death. One-year follow-up was complete in all patients. Of note, 476 (79%) patients had longer follow-up. The median follow-up duration was 629 (584–746) days.

### Endpoints

The present post-hoc analysis of the prospective HAWKEYE study [7] investigated the relationship between the TSG post-PCI and clinical outcome at vessel level. The primary endpoint was the vessel-oriented composite endpoint (VOCE), defined as the composite of vessel-related cardiovascular death, vessel-related myocardial infarction (MI) not related to the index PCI procedure, and ischemia-driven target vessel revascularization (TVR) throughout long-term follow-up. We also evaluated VOCE at one year. Secondary endpoints were (i) cumulative occurrence of vessel-related cardiovascular death and MI and (ii) cumulative occurrence of ischemia-driven TVR. All events were adjudicated by an independent clinical event committee, blinded to QFR and QCA values. Events were designated as vessel-related or not vessel-related. All deaths were considered cardiac unless an unequivocal non-cardiac cause could be established. Cardiovascular death in patients with multiple treated vessels was assigned to each vessel [7, 28]. The diagnosis of MI, as described by the Fourth Universal Definition of MI [29], required a combination of symptoms, ECG changes and significant increase in cardiac markers (troponin). Any MI without clearly identifiable culprit vessel was counted as target vessel-related. Ischemia-driven TVR was defined as any repeated revascularization of the target vessel in presence of a lesion with %DS > 50% and concomitant history of angina pectoris plus objective signs of ischemia at rest or during exercise test (or equivalent) or abnormal results of any invasive functional diagnostic test. In case of repeated adverse events on the same vessel, the first occurred was the one considered for analysis.

### Statistical analysis

Descriptive statistics were performed on the overall population grouped by the study outcome. Continuous variables are presented as mean (with standard deviation) or median [with interquartile range (IQR)], according to their distribution, and categorical variables as counts and proportions (%). For continuous variables, the differences were compared between groups using the Student t-test and the Wilcoxon test for parametric and non-parametric data, respectively. Fisher exact or Pearson Chi-squared test, with Yate’s correction when appropriate, were employed for categorical variables comparisons. Youden’s index calculation was employed to identify the optimal cut off for the QFR-TSG variable that was associated with outcome; an indicator variable was generated according to it for the subsequent analysis. Cox proportional hazards regression model with robust variance to account for patient’s correlation was used to analyze the effect of baseline and prognostic variables toward the relative risk of death. Tests for proportional hazard of each variable were based on the scaled Schoenfeld residuals. We used Kaplan–Meier plots to display the cumulative risk of VOCE over time in each treatment group. We used Cox models to estimate mortality hazard ratios (HR) comparing the four study groups based on vessel QFR and TSG values.

Association between all baseline variables and in-hospital mortality was tested in univariable regression model and those variables found to be significant (p < 0.1) were included in adjusted multivariate Cox regression analysis. The multicollinearity was examined using the variance inflation factor (VIF) and variables with VIF > 3 were excluded by the same multivariable model. The final reduced model was obtained by backward stepwise variable selection performed with Bayesian information criterion (BIC) minimization. Results were reported as hazard ratios with associated 95% confidence intervals (CIs). Receiver operating characteristic (ROC) for the Cox model was plotted and AUC was calculated and reported together with 95% confidence interval and significance. All analysis were carried out by and independent statistician (MM) with R 3.6 (R Core Team. 2020. R Foundation for Statistical Computing. Vienna. Austria) and STATA 17 (StataCorp. 2021. College Station, TX, StataCorp LLC).

## Results

The HAWKEYE study evaluated 751 vessels in 602 patients enrolled from June 2016 to July 2017 (6). The median value of QFR-TSG of all vessels was 0.00 [IQR 0.00–0.01]. Based on post-PCI QFR, with 0.90 as cut-off value to define high or low post-PCI QFR, and QFR-TSG, divided at the median value, vessels were divided into four groups. More than half of the vessels (412 [54.8%]) had a post-PCI QFR ≥ 0.90, and a QFR-TSG = 0 (group 1). Group 2 was constituted by vessels with QFR ≥ 0.90 and QFR-TSG > 0 (n = 216 vessels, 28.7%). Group 3 was composed of post-PCI QFR < 0.90 and QFR-TSG = 0 (n = 37, 4.9% of entire population). Finally, 86 (11.4%) vessels had low post-PCI QFR and high QFR-TSG (group 4). Overall, 449 vessels (59.8%) vessels had a QFR-TSG = 0 compared to 302 (40.2%) vessels with a QFR-TSG > 0 (Table 1 and 2).

**Table 1:** Baseline and procedural characteristics at the patient and vessel level

Patient level	Group 1 293 (48)	Group 2 194 (32)	Group 3 34 (6)	Group 4 81 (14)	p value
Age, years	68 [59–77]	69 [60–77]	67 [60–77]	68 [60–79]	0.79
Female sex, no. (%)	84 (28)	50 (26)	6 (18)	19 (23)	0.62
CV risk factors, no. (%)					
Diabetes	60 (21)	54 (28)	4 (12)	21 (26)	0.09
Hypertension	201 (69)	146 (75)	28 (82)	69 (85)	0.01
Hyperlipidemia	152 (52)	116 (60)	18 (53)	50 (62)	0.22
Current smoker	48 (16)	40 (21)	10 (29)	16 (20)	0.25
Medical history, no. (%)					
MI	52 (18)	54 (28)	8 (24)	19 (23)	0.07
PCI	60 (21)	59 (30)	8 (24)	20 (25)	0.09
CVA	2 (0.7)	3 (1.5)	1 (2.9)	3 (3.7)	0.21
PAD	16 (5.4)	15 (7.7)	2 (5.9)	6 (7.4)	0.76
Chronic kidney disease *	23 (7.8)	16 (8.2)	0 (0)	9 (11)	0.52
Clinical presentation, no. (%)					
NSTEACS	197 (67)	129 (66)	23 (68)	53 (65)	0.99
SIHD	96 (33)	65 (34)	11 (32)	28 (35)	0.99
Angiographic disease severity					
SYNTAX score, point	9 [5–16]	15 [7–22]	14 [9–23]	15 [7–25]	0.00001
*Medical therapy, no*. (%)					
ASA	290 (98)	187 (96)	33 (97)	78 (96)	0.99
Clopidogrel	121 (41)	76 (39)	14 (41)	32 (39)	0.96
Ticagrelor	140 (47)	94 (48)	16 (47)	39 (48)	0.98
DAPT	261 (89)	170 (87)	30 (88)	71 (87)	0.97
Statins	230 (78)	148 (76)	26 (76)	64 (79)	0.89

Vessel level	Group 1 412 (55)	Group 2 216 (29)	Group 3 37 (5)	Group 4 86 (11)	p value
Location, no (%)					
LAD	242 (59)	150 (69)	31 (84)	70 (81)	0.00001
LCx	170 (41)	76 (35)	8 (22)	30 (35)	0.07
RCA	167 (40)	78 (36)	14 (38)	28 (33)	0.47
Quantitative coronary angiography					
Pre-PCI RVD, mm	2.8 [2.3–3.2]	3 [2.2–3.6]	2.7 [2.2–3.1]	2.8 [2.4–3.1]	0.37
MLD, mm	1.7 [1.5–2.1]	1.9 [1.6–2.3]	1.6 [1.4–2]	1.7 [1.4–1.9]	0.31
Pre-PCI diameter stenosis, %	62 [53–76]	64 [53–77]	58 [53–74]	59 [51–67]	0.86
Pre-PCI lesion length, mm	9 [8–19]	20 [11–32]	21 [10–31]	23 [11–34]	< 0.00001
Post-PCI diameter stenosis, %	8 [7–16]	12 [10–23]	7 [6–19]	13 [11–24]	< 0.00001
Procedural data					
Number of stents, no	1 [1, 2]	1 [1, 2]	1 [1, 2]	1 [1, 2]	0.10
Diameter of stents, mm	3 [2.5–3.3]	3 [2.6–3.4]	3 [2.7–3.4]	2.9 [2.5–3.2]	0.83
Total length of stents, mm	15 [14–31]	22 [21–43]	17 [16–35]	27 [16–43]	< 0.00001
Postdilation, no. (%)	518 (87)	29 (88)	77 (85)	31 (91)	0.87

^*^Defined as creatinine ≥ 2 mg/dl

*BMI* body mass index, *CV* cardiovascular, *MI* myocardial infarction, *PCI* percutaneous coronary intervention, *CVA* cerebrovascular accident, *PAD* peripheral artery disease, *NSTEACS* non ST-segment elevation acute coronary syndrome, *SIHD* stable ischemic heart disease, *LAD* left anterior descending. *LCx* left circumflex, *RCA* right coronary artery, *RVD* reference vessel diameter, *MLD* minimal lumen diameter, *TSG* trans stent gradient

**Table 2:** Occurrence of the VOCE, TV-MI and TLF in long-term follow-up stratified according to vessel QFR and TSG

Subgroups	Vessels (n = 751)	VOCE	p	TV-MI	p	TLF	p
Group 2, no (%)	216 (28.7)	16 (7.4)		9 (4.1)		13 (6)	
Group 3, no (%)	37 (4.9)	4 (10.8)		2 (5.4)		3 (8)	
Group 4, no (%)	86 (11.4)	27 (31.4)		8 (9.3)		21 (24.4)	
TSG = 0, no (%)	449 (59.8)	10 (2.2)	< 0.01	5 (1.1)	< 0.01	6 (1.3)	< 0.01
TSG > 0, no (%)	302 (40.2)	43 (14.2)		17 (5.6)		34 (11.3)	

Group 1: post-PCI QFR ≥ 0.90 and TSG = 0. Group 2: PCI QFR ≥ 0.90 and TSG > 0. Group 3: PCI QFR < 0.90 and TSG = 0. Group 4: post-PCI QFR < 0.90 and TSG > 0

*VOCE* vessel oriented composite endpoint, *QFR* quantitative flow ratio, *TSG* trans-stent gradient, *TV*-*MI*,target vessel myocardial infarction, *TLF* target lesion failure

Baseline characteristics were substantially well balanced between the four groups, with the exception of hypertension and SYNTAX score, which was significantly different among the four groups, with less hypertensive patients and the lowest Syntax score in Group 1. (Table 1).

Regarding QCA analysis and procedural data, there was no difference in reference vessel diameter (RVD) and pre-PCI diameter stenosis (DS). Interestingly, vessels with high QFR-TSG had longer lesions and consequently a significantly higher total stent length. Post-PCI DS was significantly greater in vessels with QFR-TSG > 0, despite no difference in terms of post-dilation, which was performed in most cases as encouraged by protocol. Detailed patient, vessel, and procedural characteristics are reported in Table 1. No significant differences in terms of antiplatelet and statin therapy were appreciated in the 4 study groups (Table 1).

### Clinical follow-up

Overall, 77 events were detected in 53 treated vessels (7%) during follow-up. In detail, we observed 11 cardiovascular deaths, 21 target vessel myocardial infarctions (TVMI) and 40 target vessel revascularizations (TVR). In the overall population we reported 5 cases (0.7%) of late stent thrombosis (ST). In particular ST occurred in 1 case of Group 1 (0.2%) and Group 4 (1.2%) and in 3 cases of Group 2 (1.4%). No cases of ST occurred in the Group 3.

**Fig. 1:**
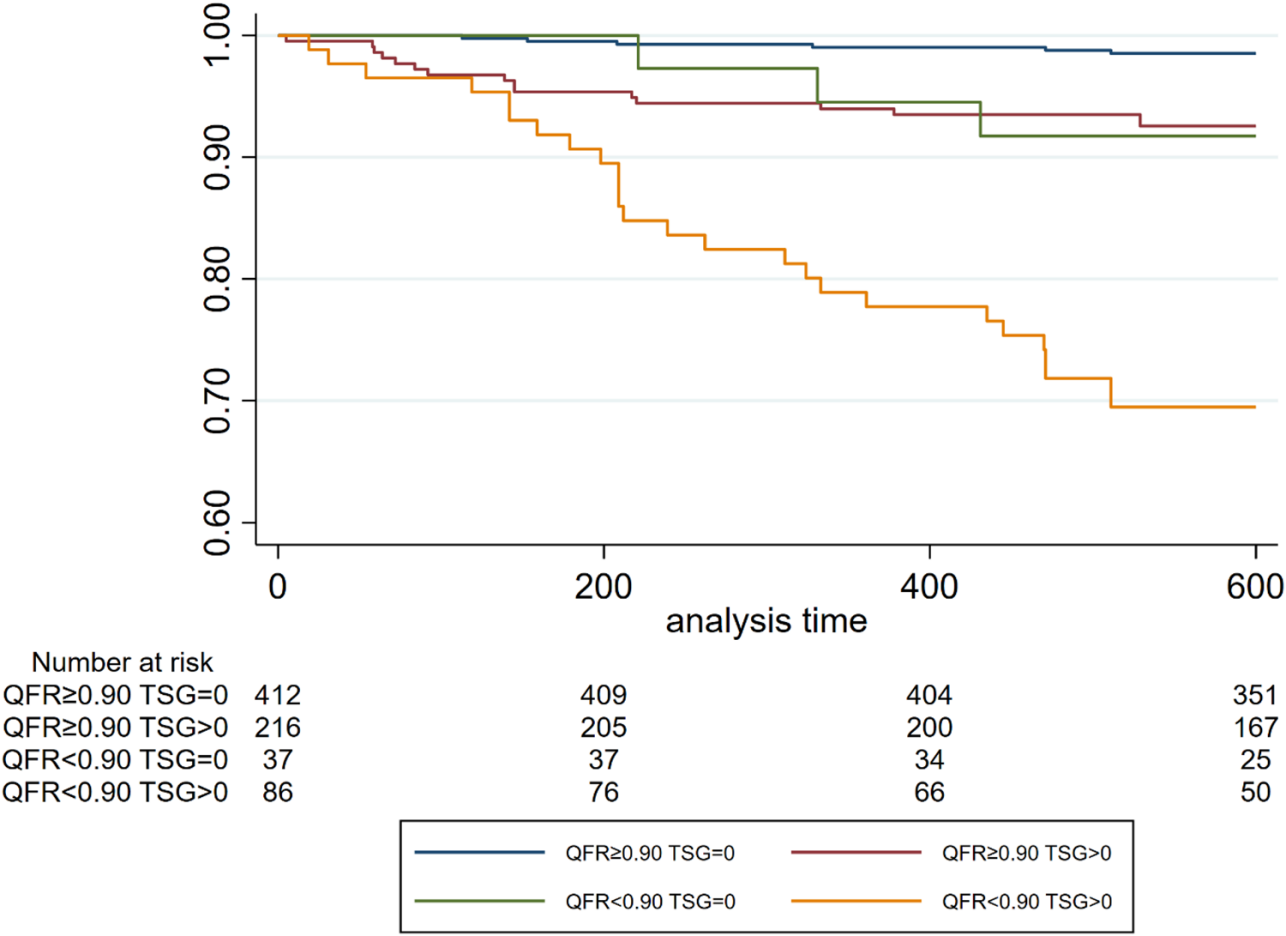
Kaplan–Meier curves displaying cumulative VOCE risk according to vessel-QFR and TSG values. *VOCE* vessel oriented composite endpoint, *QFR* quantitative flow-ratio, *TSG* trans-stent gradient

Post-PCI QFR-TSG was significantly higher in vessels with VOCE during follow-up compared with those without (0.01 [IQR: 0.01–0.05] vs. 0.00 [IQR: 0.00–0.01], respectively; p < 0.001). The occurrence of VOCE stratified according to QFR and TSG values is shown in Fig. 1. In particular, only 10 (2.2%) VOCE occurred in vessels with QFR-TSG = 0 (Groups 1 and 3), compared with 43 (14%) VOCE in vessels with high QFR-TSG (groups 2 and 4, p < 0.01, Table 2).

**Fig. 2:**
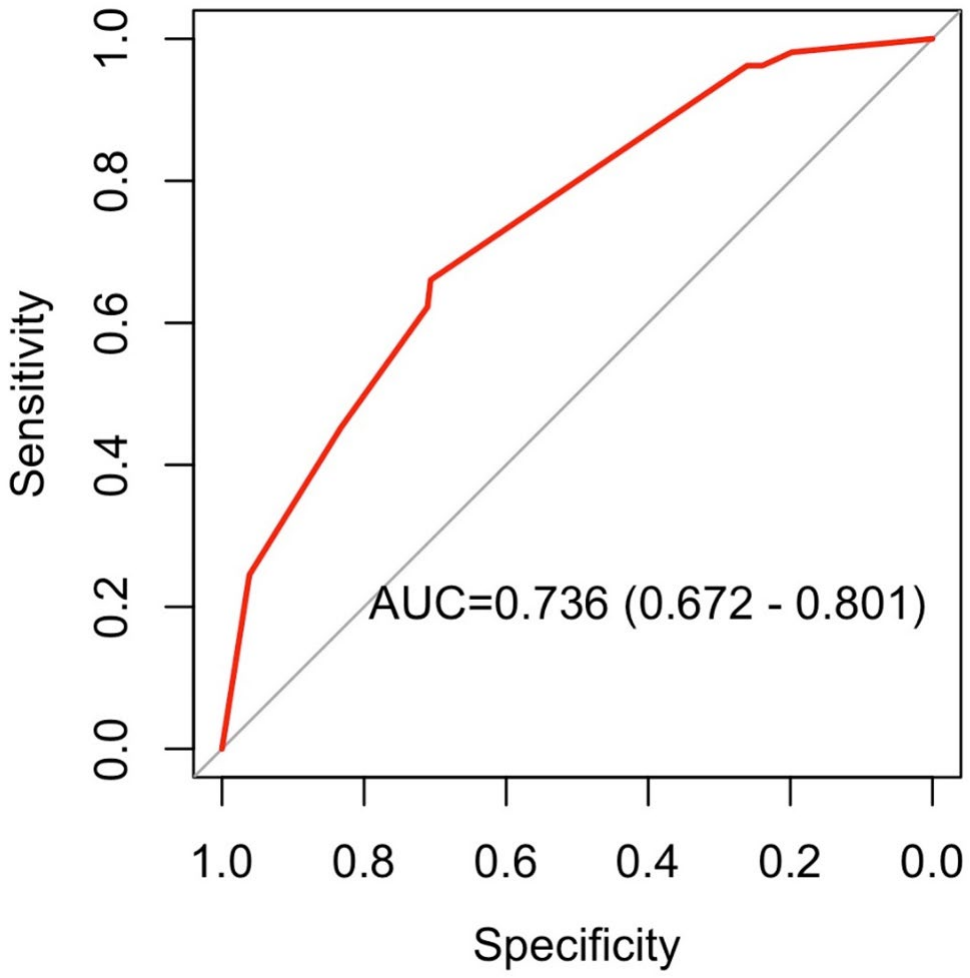
Receiver-operating characteristic curve analysis of QFR-TSG ability to predict the occurrence of VOCE in the study population. *VOCE* vessel oriented composite endpoint, *QFR* quantitative flow-ratio, *TSG* trans-stent gradient, *AUC* area under the curve

The best cut-off for QFR-TSG in our population was 0.01 (Youden Index 0.44, accuracy 0.64, sensitivity 0.81, specificity 0.63, prevalence 0.07). Receiver-operating characteristic curve analysis showed an area under the curve of 0.74 (95% CI: 0.67 to 0.80; p < 0.001, Fig. 2). In the multivariate analysis, QFR-TSG (binary threshold 0.01), along with hypertension and prior-MI, was confirmed as an independent predictor of the VOCE with an hazard ratio of 2.95 [1.77–4.91] (Table 3).

**Table 3:** Univariate and multivariable Cox regression analysis – patient level

Patients (n = 602)	Univariate	Multivariate
	HR (95%CI)	HR (95%CI)
Multivessel PCI	3.031 (1.287 – 7.14)	–
Hypertension	4.249 (1.523 – 11.853)	3.183 (1.123–9.019)
Diabetes	2.273 (1.209 – 4.276)	–
Prior MI	3.694 (2.029 – 6.727)	3.051 (1.681–5.538)
Prior PCI	2.919 (1.594 – 5. 347)	–
Prior CVA	0.018 (0.009 – 0.037)	–
Number of stents implanted	1.299 (1.004 – 1.679)	–
Vessel cQFR	0.5 (0.421 – 0.594)	–
Lesion cQFR	0.877 (0.849 – 0.905)	–
TSG	1.141 (1.105 – 1.178)	2.949 (1.771–4.91)
Post-PCI %DS	1.788 (1.45 – 2.204)	–
Post-PCI stent length	1.248 (1.002 – 1.553)	–
Syntax score	1.785 (1.21 – 2.633)	–
cQFR ≤ 0.89	8.016 (4.676 – 13.741)	–

The analysis is for the prediction of the composite endpoint of CV death, TVMI and TVR

*PCI* percutaneous coronary intervention, *MI* myocardial infarction, *CVA* cerebrovascular accident, *cQFR* contrast quantitative flow ratio, *TSG* trans-stent gradient, *DS* diameter stenosis

**Fig. 3:**
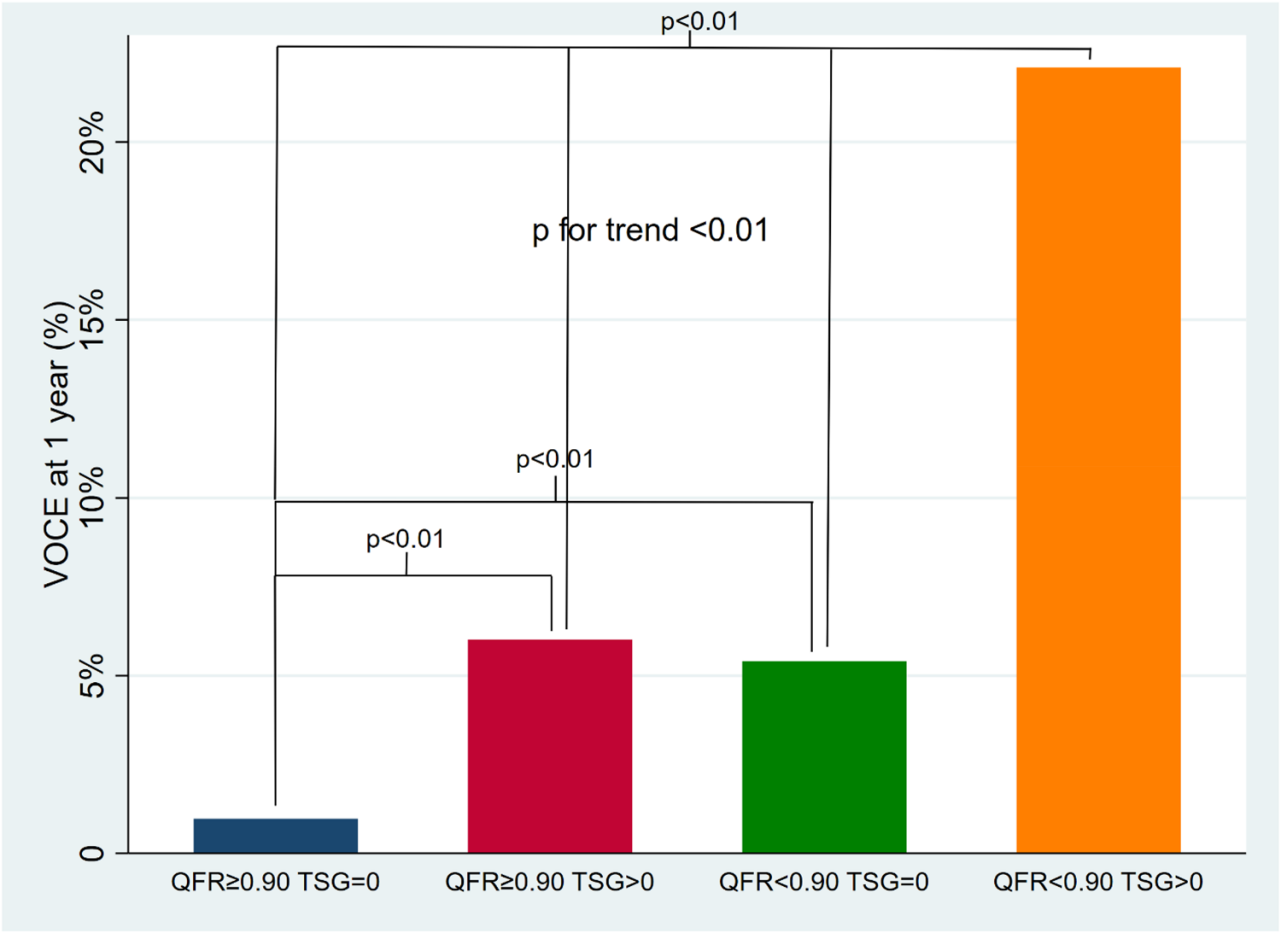
Graph bar showing the percentage of VOCE at 1-year according to QFR and TSG values. *VOCE* vessel oriented composite endpoint, *QFR* quantitative flow-ratio, *TSG* trans-stent gradient

Figure 1 shows the Kaplan–Meier curves stratified according to QFR and TSG values. As expected, vessels with high post-PCI QFR (≥ 0.90) and QFR-TSG = 0 (group 1) had a very low rate of events. (Fig. 1). Groups 2 and 3 had a comparable rate of events (Fig. 1).Patients in Group 4 with low FFR and high TSG had the highest MACE rate (30%). Similar results are shown in the 1-year analysis (Fig. 3).

## Discussion

The HAWKEYE study was conducted to investigate the potential role of QFR after successful PCI with stent implantation in the prediction of adverse events [7]. A post-PCI QFR below 0.90 was associated with worse clinical outcome [7]. The analysis of the location of a drop in QFR showed different mechanisms underlying lower post-PCI QFR value: (i) residual diffuse disease, (ii) focal drop outside the stent, and iii) focal drop within the stented segment [8].

The present analysis of the HAWKEYE study was designed to measure in all vessels the gradient of QFR across the stented segment. The major findings include the following:

- In angiographically optimized vessels after PCI the drop within the stented segment measured with QFR is either low or absent and appears lower than the value previously reported with FFR (24,25);
- approximately 40% of vessels had a QFR-TSG > 0;
- patients with QFR-TSG ≥ 0.01 had a worse clinical outcome in terms of VOCE, irrespectively of the vessel QFR value;
- QFR-TSG was an independent predictor of VOCE, confirmed in multivariate analysis.
- The combination of high TSG and low QFR had a markedly worse outcome than other groups.

Functional assessment after stent implantation can provide useful information regarding the success of coronary revascularization [5, 7]. FFR represents the “gold standard” in the field of intracoronary physiology. In recent years several other tools have demonstrated efficacy in evaluating functional value of coronary lesions, such as resting invasive indices and angiographically-based functional assessment (QFR). These tools can be used to both guide and optimize revascularization. Recently, a flowchart to guide the functional optimization of PCI using those different methods has been provided [5]. Regardless of the utilized tool, the crucial concept of functional optimization is to localize the residual disease burden after PCI, in order to correct it if possible. When an FFR pullback was systematically performed after PCI, a significant pressure drop inside the stented segment, with a value of 0.04 or more, was present approximately 40% of stented segments and it was a predictor of a lower post-PCI FFR and poorer outcome [24, 25]. Thus, the value in knowing the trans-stent gradient is important as it is potentially correctable cause of high TSG and low FFR.

In our study, the computation of a QFR gradient across the stent was feasible and its presence was associated with a worse outcome. Despite angiographically procedural success as defined, approximately 40% of vessels had a pressure drop across the stent (QFR-TSG > 0) suggesting relative stent underexpansion or undersizing or possibly other mechanisms in individual vessels. A post-PCI QFR-TSG > 0 was associated with worse clinical outcome, even in vessels with a QFR ≥ 0.90. This finding is important because a drop within the stent may be treatable through further post-dilation or with imaging evaluation to determine the specific cause of the elevated TSG.

One of the main advantages of QFR is that it does not require passing a wire into a freshly stented area or utilizing a pressure wire that has been in place for the entire PCI which may “drift” over that period. With the use of dedicated software, functional evaluation with QFR can be determined rapidly after stent implantation, with a virtual pullback providing further information about possible sources of any residual pressure drop [5, 7, 8].

Combining the results of the initial HAWKEYE study [7] and this post-hoc HAWKEYE analysis, the emerging concept is that the ideal post-PCI functional assessment should always include not only the entire vessel QFR computation but also the measure of gradient within the stented segment. Indeed, patients with a post-PCI QFR ≥ 0.90 but with a TSG > 0 had similar outcomes compared to those with a vessel QFR < 0.90 but without any TSG. Hence, also in patients with post-PCI QFR value ≥ 0.90, we may improve clinical outcome with an optimization of PCI in case of detection of a significant gradient across the stent. These findings are consistent with a large body of studies demonstrating consistently that stent underexpansion is a major determinant of late adverse outcomes [5, 7, 8].

What is unknown is whether improving a high TSG after stenting will favourable affect long-term outcomes. A randomized trial comparing angiography alone vs QFR post-PCI is needed to determine the clinical value of this approach.

### Study limitations

The present study has several important limitations. First, it is a post-hoc analysis of a prospective study, which was not designed for the current aim. Second, the number of patients and events in the four groups are relatively small and the presence of some differences among the four groups cannot be ruled out. That being said, it represents a fairly large patient group with complete long-term follow-up. It is the largest current study evaluating the outcomes relative to the level of TSG. Third, in the HAWKEYE study [7], post-PCI FFR was not invasively measured. Thus, we cannot provide any direct comparison between TSG FFR and QFR, although previous studies have demonstrated an excellent correlation between FFR and QFR [26, 27]. In addition, when present, TSG was numerically very low. Therefore, the reproducibility of our results in clinical settings should be confirmed by further studies. Finally, it is also important to remind that the most frequent underlying mechanism of suboptimal physiology after PCI is related to untreated lesions outside the stent. However, TSG emerged as independent predictor of adverse events, irrespectively from the vessel-QFR value.

## Conclusions

The measurement of post-PCI QFR-TSG after successful PCI with stent implantation is feasible. An increase QFR-TSG was an independent predictor of adverse events and identified a subgroup of patients at higher risk for poor outcomes. The combination of high QFR and low TSG demonstrated the best long-term outcome whereas low QFR and high TSG showed the worst outcome.

## Supplementary Information

Below is the link to the electronic supplementary material.

Supplementary file1 (DOCX 4243 kb)
