# Supplementary material for: Impact of trans-stent gradient on outcome after PCI: results from a HAWKEYE substudy
Source: Int J Cardiovasc Imaging. 2022 Aug 22;38(12):2819–27. doi: 10.1007/s10554-022-02708-7 (PMC9708807; doi:10.1007/s10554-022-02708-7)
Supplement: Supplementary file 1 — Supplementary file1 (DOCX 4243 kb) [file 10554_2022_2708_MOESM1_ESM.docx]

**Supplementary Materials**

**Page 2: Supplemental Figure 1:** Histogram of TSG distribution

**Page 3: Supplementary methods:** Step by step explanation of TSG calculation

**Page 4: Explicative examples per group**

**Histogram of TSG distribution**

**
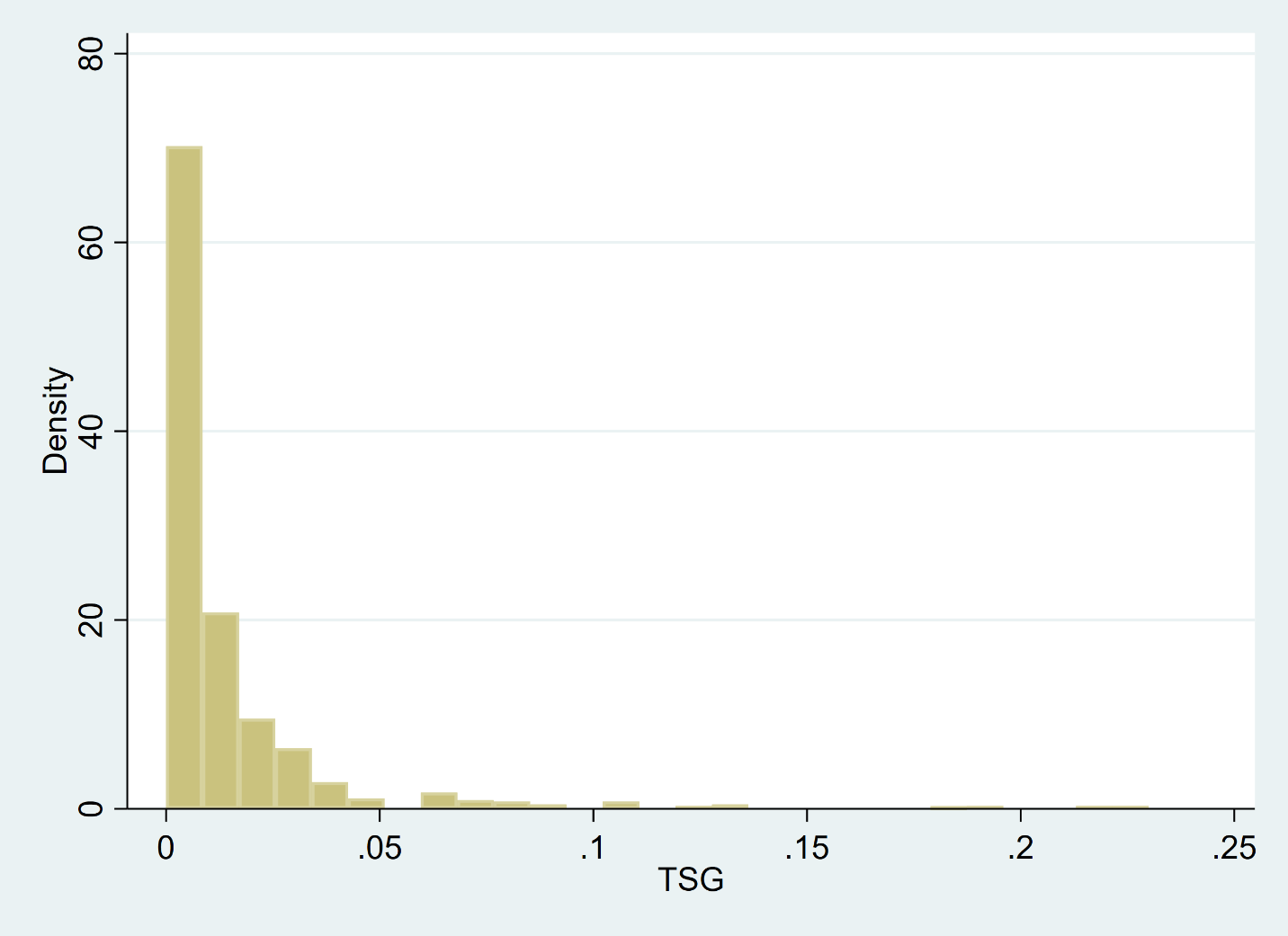
**

TSG: trans-stent gradient.

**Supplementary Methods**

**Step by step explanation of TSG calculation**


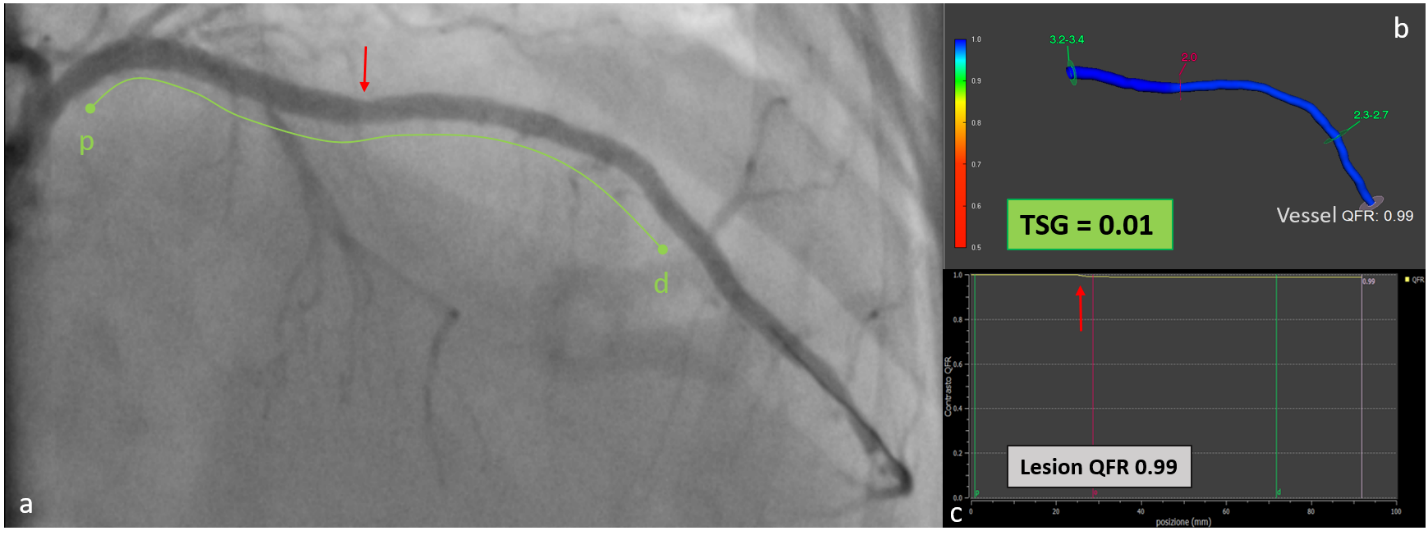
Green line in the panel a indicates the stented segment of the vessel, with p and d as the proximal and distal markers of the stent edges.

Panel b represents the 3D reconstruction of the vessel, with the final QFR result = 0.99.

Panel c represents the virtual pull-back of functional assessment (yellow line). P and d (green lines) indicate the proximal and distal markers of the stented segment. Lesion QFR is the value of QFR calculated inside the p and d markers, and so across the stented segment. Red arrow indicates the exact point of pressure drop along the vessel.

The TSG is calculated by subtracting the lesion QFR value by 1.00, that is the ideal QFR value with perfect result after stenting and without any residual disease. TSG = 1 – 0.99 (lesion QFR) = 0.01.

**Explicative example for group 1:**


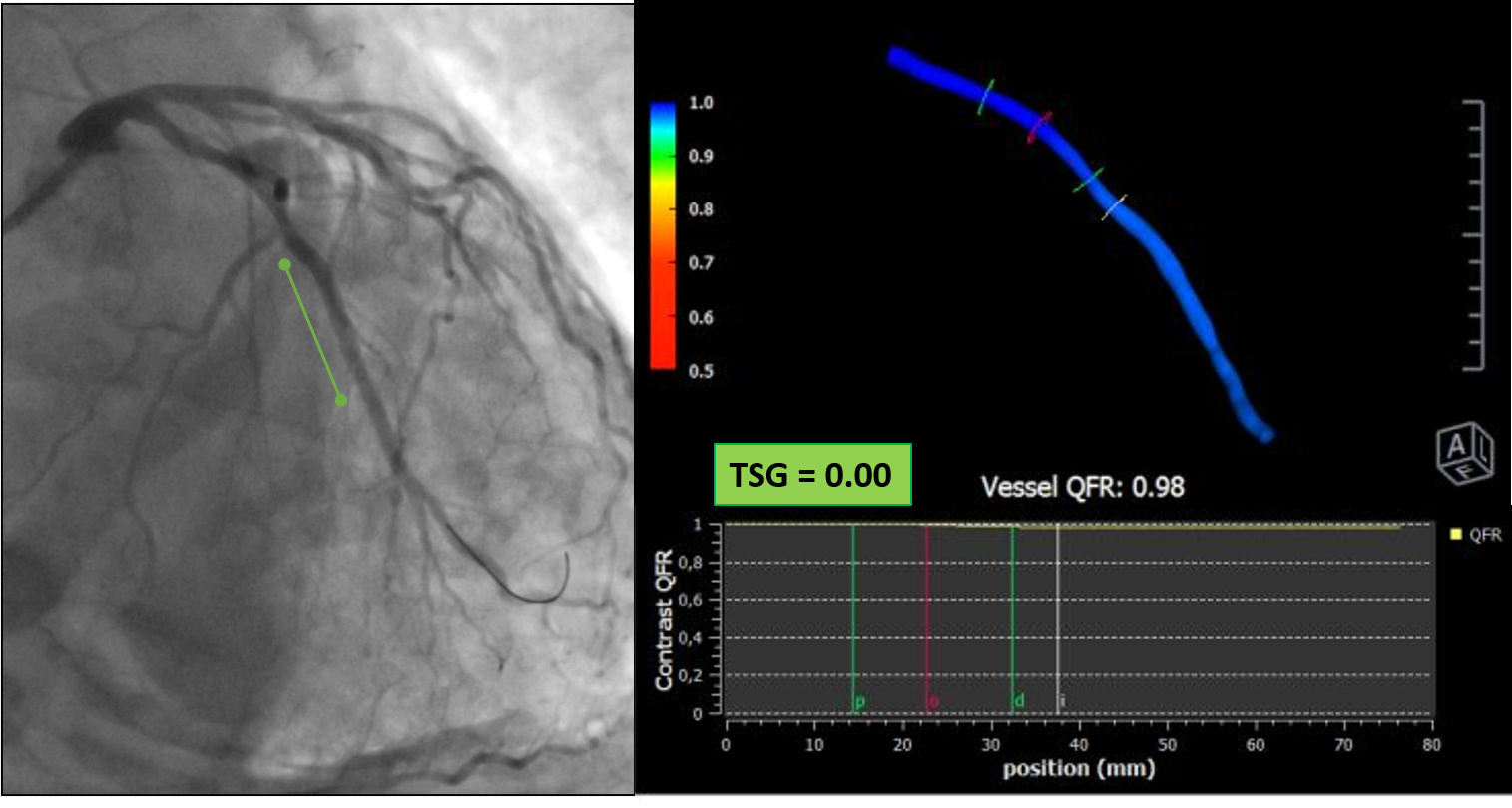


Good result across the stented segment (indicated by green line) without trans-stent gradient (TSG = 0.00) and concordant good result of the entire vessel (vessel QFR post-PCI > 0.90).

**Explicative example for group 2**

**
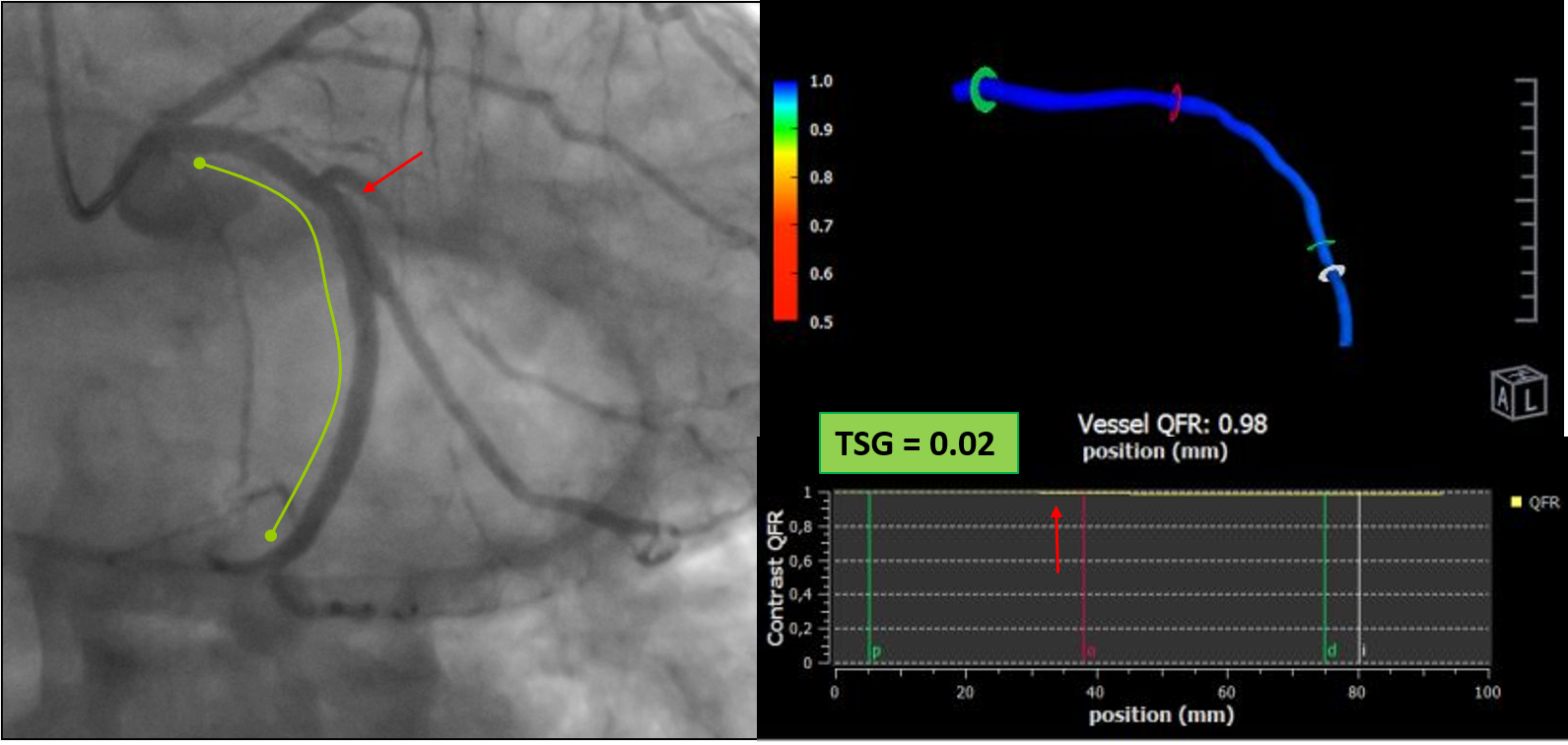
**

Good result of the entire vessel (vessel QFR post-PCI > 0.90) with the presence of a small trans-stent gradient (TSG = 0.02) due to minimal unexpanded area across stented segment (indicated by green line and red arrow).

**Explicative example for group 3:**


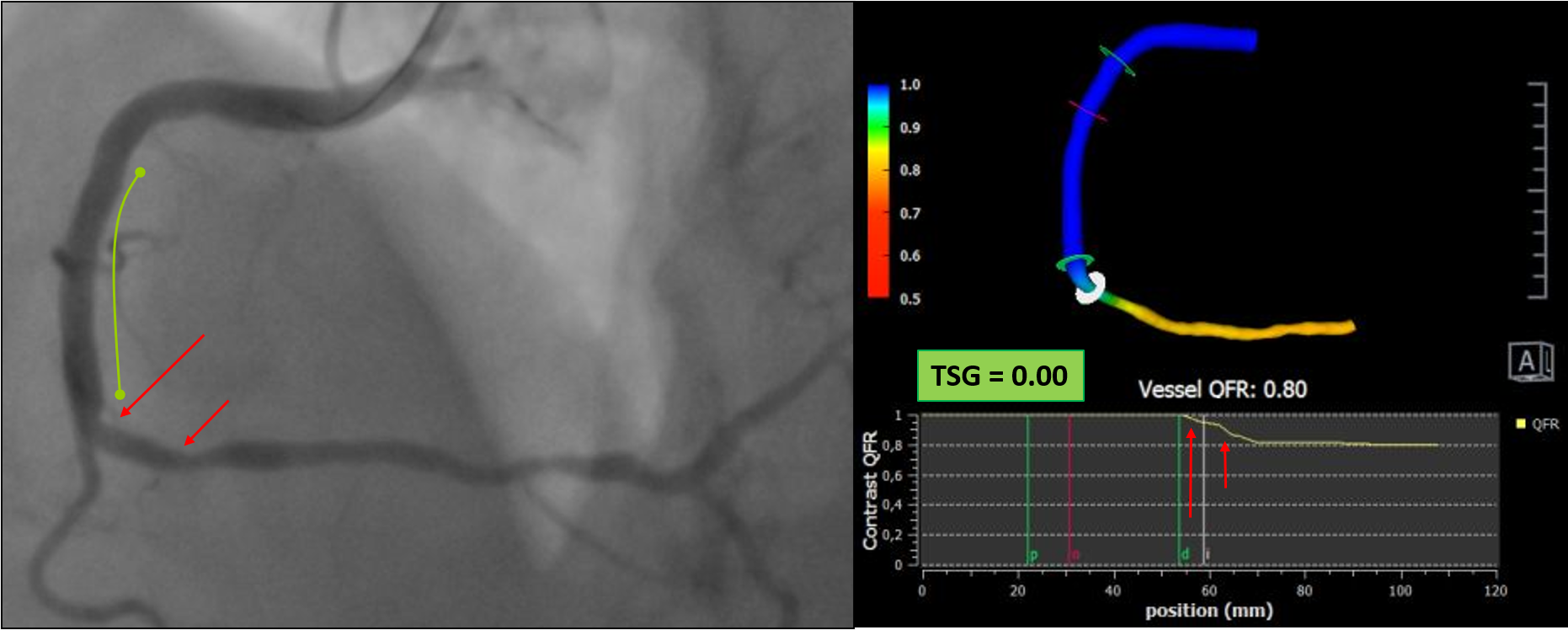


Good result across the stented segment (indicated by green line) without trans-stent gradient (TSG = 0.00) with poor result of the entire vessel (vessel QFR post-PCI < 0.90) due to two unnoticed lesions out of stented segment (indicated by red arrows) that were left untreated.

**Explicative example for group 4:**


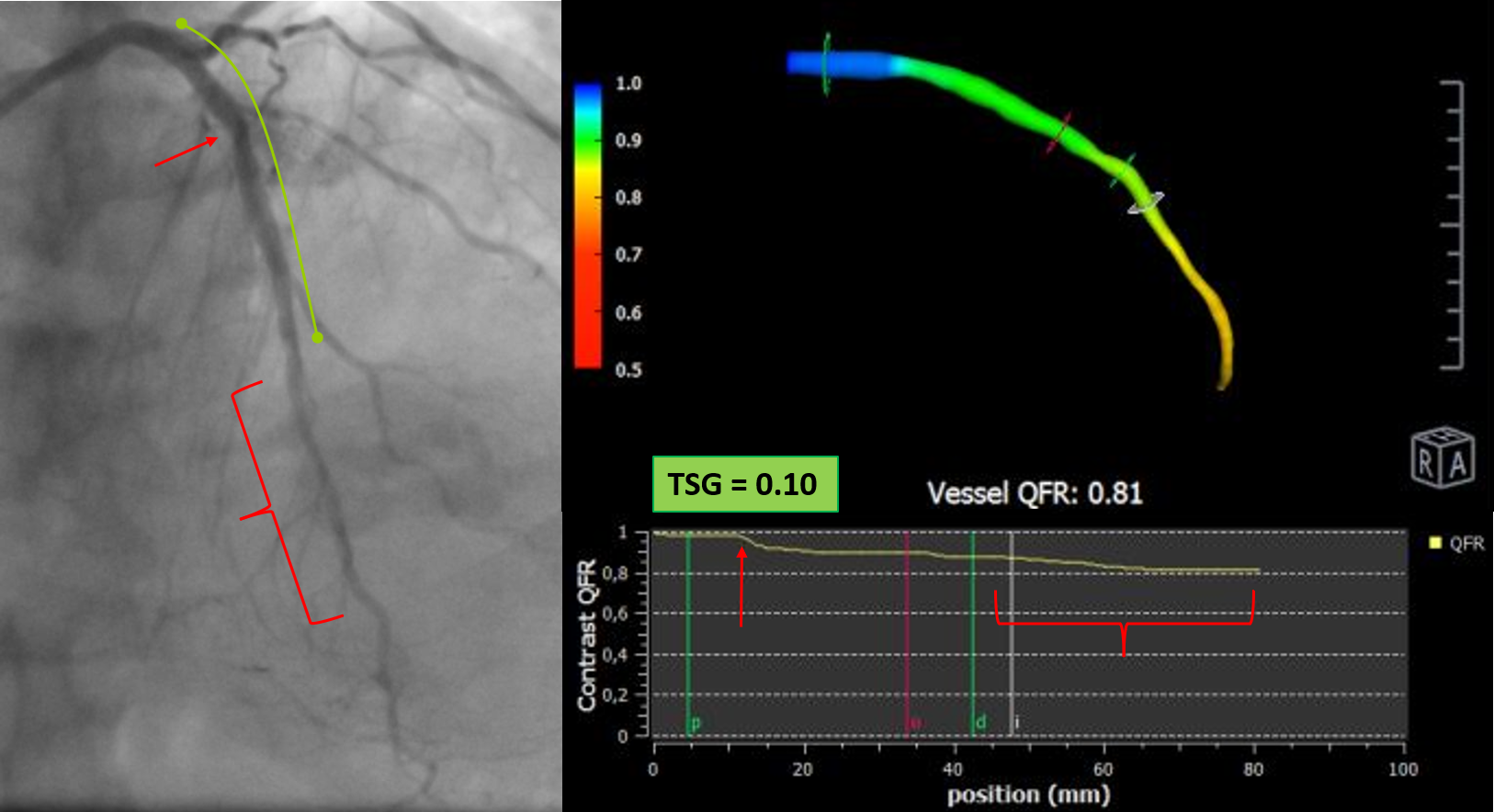


Suboptimal result of the entire vessel (vessel QFR post-PCI < 0.90) due to residual diffuse disease out of stented segment (indicated by red bracket) and also a poor result across the stented segment (indicated by green line) with a significant trans-stent gradient (TSG = 0.10) due to unexpanded area (indicated by red arrow).
